# Accumulation of Carbonyl Proteins in the Brain of Mouse Model for Methylglyoxal Detoxification Deficits

**DOI:** 10.3390/antiox10040574

**Published:** 2021-04-08

**Authors:** Shin Koike, Kazuya Toriumi, Sakura Kasahara, Yosuke Kibune, Yo-ichi Ishida, Takashi Dan, Toshio Miyata, Makoto Arai, Yuki Ogasawara

**Affiliations:** 1 Department of Analytical Biochemistry, Meiji Pharmaceutical University, Tokyo 204-8588, Japan; skoike@my-pharm.ac.jp (S.K.); m196206@std.my-pharm.ac.jp (S.K.); buyobuyo3987@gmail.com (Y.K.); 2 Schizophrenia Research Project, Department of Psychiatry and Behavioral Sciences, Tokyo Metropolitan Institute of Medical Science, Tokyo 156-8506, Japan; toriumi-kz@igakuken.or.jp (K.T.); arai-mk@igakuken.or.jp (M.A.); 3 Department of Microbial Science and Host Defense, Meiji Pharmaceutical University, Tokyo 204-8588, Japan; ishida@my-pharm.ac.jp; 4 Division of Molecular Medicine and Therapy, Tohoku University Graduate School of Medicine, Sendai 980-8575, Japan; dantks@med.tohoku.ac.jp (T.D.); miyata@med.tohoku.ac.jp (T.M.)

**Keywords:** carbonyl stress, pyridoxamine, scavenger, schizophrenia, methylglyoxal-induced oxidative damages, mitochondrial creatine kinase

## Abstract

Recent studies have shown that carbonyl stress is a causative factor of schizophrenia, categorized as carbonyl stress-related schizophrenia (CS-SCZ). However, the correlation between carbonyl stress and the pathogenesis of this disease is not well established. In this study, glyoxalase 1(Glo1)-knockout and vitamin B6-deficient mice (KO/VB6 (-) mice), which are susceptible to methylglyoxal (MGO)-induced oxidative damages, were used as a CS-SCZ model to analyze MGO-modified protein and the carbonyl stress status in the brain. A comparison between Wild/VB6(+) mice and KO/VB6(−) mice for accumulated carbonyl proteins levels, with several advanced glycation end products (AGEs) in the brain, revealed that carbonyl protein levels with the Nδ-(5-hydro-5-methyl-4-imidazolon-2-yl) ornithine (MG-H1) moiety were significantly increased in the hippocampus, prefrontal cortex, striatum, cerebral cortex, and brainstem regions of the brain in KO/VB6(−) mice. Moreover, two-dimensional electrophoresis and Liquid chromatography-tandem mass spectrometry analysis showed MG-H1-modified arginine residues in mitochondrial creatine kinase, beta-adrenergic receptor kinase 1, and T-complex protein in the hippocampus region of KO/VB6(−) mice, but not in Wild/VB6(+) mice. In particular, MG-H1 modification of mitochondrial creatine kinase was quite notable. These results suggest that further studies focusing on MG-H1-modified and accumulated proteins in the hippocampus may reveal the onset mechanism of CS-SCZ induced by MGO-induced oxidative damages.

## 1. Introduction

Schizophrenia (SCZ) causes severe mental disability and affects approximately 1% of the global population [1]. Although several studies have been conducted to understand the underlying mechanism of the disease, the main cause and pathophysiology of SCZ remains elusive, in part due to considerable heterogeneity in its symptoms and the long-term clinical courses between patients. To understand the onset mechanism, we focused on the involvement of carbonyl stress-related schizophrenia (CS-SCZ), which was recently discovered in a population of patients with refractory SCZ [2,3,4,5,6]. Carbonyl stress is an abnormal metabolic state resulting from either increased production of reactive carbonyl compounds (RCOs), such as methylglyoxal (MGO), or decreased detoxification of RCOs [7]. RCOs react with amino acid moieties of proteins to form carbonyl proteins, which eventually lead to the formation of advanced glycation end products (AGEs) through a complex mechanism. Carbonyl proteins or AGEs have been implicated in a variety of diseases, including diabetes mellitus [8,9,10], chronic kidney diseases [7], and cardiovascular diseases [11]. We previously reported that a significant number of patients (approximately 20%) with SCZ showed high levels of blood pentosidine (PEN), a typical AGE, or low levels of vitamin B6 (VB6), which is known to scavenge free carbonyl groups [2,12]. We also reported that the administration of pyridoxamine, a carbonyl scavenger, in patients with refractory SCZ improved its symptoms in some cases [13]. MGO, a carbonyl compound, is presumed to be a causative factor of CS-SCZ. Many reports have suggested that MGO, a by-product of the glycolytic pathway, is responsible for diabetic complications and cardiovascular diseases [14,15]. However, few researchers have analyzed the role of MGO in mental and neurological disorders. Decreased expression of glyoxalase 1(Glo1), a gene related to MGO and glyoxal detoxification, is reported to promote anxious behavior in mice [16]. This suggests that glucose metabolism disorders or carbonyl compounds may be involved in the pathogenesis of CS-SCZ. Furthermore, a recent study reported that MGO-mediated anxiolysis leads to an increase in protein modification and an elevated expression of Glo1 in the brain [17]. However, there have been no detailed studies on protein carbonylation within the brain and its correlation with CS-SCZ. Therefore, in order to understand the onset mechanism of this disease, we attempted to establish a novel mouse model combined with Glo1-knock out and vitamin B6 deficiency (KO/VB6(−) mice) as a CS-SCZ model, which lack the ability to scavenge excess methylglyoxal. In this study, we expected that specific proteins were excessively carbonylated under impairment of MGO detoxification systems, and schizophrenia-like behavioral deficits might be caused via dysfunction of the proteins carbonylated by MGO. To prove the hypothesis, proteomic analysis was carried out to investigate the accumulation of AGE-modified proteins and identify the specifically modified protein in the brain of a mouse model with MGO-induced oxidative damages.

## 2. Materials and Methods

### 2.1. Experimental Animals

Eight-week-old *Glo1*-KO mice were fed a VB6-deficit diet for 4 weeks and referred to as “CS-SCZ model mice.” In addition, 8-week-old *Glo1*-KO mice fed with a normal diet for 4 weeks, and 8-week-old C57BL/6J wild-type (WT) mice fed with a normal diet or VB6-deficit diet for 4 weeks were used as three control groups [18].

### 2.2. Generation of Glo1-KO Mice and VB6-Deficit Diet

A 129/Sv-derived Embryonic stem cell line (S17-2B1) containing a gene trap cassette in the *Glo1* gene locus was purchased from Mutant Mouse Regional Resource Centers (http://www.mmrrc.org/ accessed on 8 July 2020). ES cells were microinjected into embryonic day 3.5 (E3.5) blastocysts (genotype C57BL/6J), followed by their transfer into pseudo-pregnant ICR females for the generation of chimeras. Chimerism of newborn mice was assessed by coat color inspection. Male mice with chimera with high ES cell contribution (100% agouti coat color) were crossed with C57BL/6J mice. The germline transmission was ascertained by the presence of agouti pups (F1 generation), and disrupted alleles were identified by Polymerase Chain Reaction (PCR) genotyping, as indicated below. One pair of heterozygous (HE) F1 mice was mated to confirm that the generated F2 Glo1 KO mouse was non-lethal and normal in appearance. KO, HE, and wild-type mice (WT) were identified by PCR genotyping before being subjected to further studies. No significant increases in the gene expression of Akr family and Park7 were confirmed in Glo1-KO mice [18]. 

To induce VB6 deficiency, WT and KO mice were fed with a VB6-lacking diet containing 5 µg/100 g VB6 pellets from 8 to 12 weeks of age, while control mice in normal VB6 condition were fed with a normal diet, with 1.4 mg/100 g VB6 pellets. Food and tap water were available ad libitum.

### 2.3. Sample Preparation

Brain tissue samples were prepared as previously described [14]. Briefly, the brains were removed after perfusion with phosphate-buffered saline (PBS) and the specific brain regions, including the prefrontal cortex (PFC), hippocampus (HIP), nucleus accumbens, striatum (STR), brain stem (BS), and cerebellum, were fractionated by a routine procedure. Each brain region was homogenized using BioMasher (Nippi Inc., Tokyo, Japan) with nine volumes of ice-cold buffer (0.1 M phosphate buffer (pH 7.8) and a protease inhibitor cocktail (Nacalai tesque, Kyoto, Japan)). To prepare protein extracts, the brain homogenates were centrifuged at 12,000× *g* for 10 min at 4 °C and the supernatant was stored at −85 °C before use. 

### 2.4. Western Blot Analysis

Protein extracts from the brain regions were boiled for 3 min in a sodium dodecyl sulfate (SDS) buffer (Nacalai tesque, Kyoto, Japan) and then resolved by 5–20% (*w/v*) SDS-polyacrylamide gradient gel electrophoresis (ATTO, Tokyo, Japan), followed by transfer of the separated proteins onto an Immobilon-P polyvinylidene difluoride (PVDF) membrane (Millipore, Billerica, MA, USA). The PVDF membrane with the transferred proteins was blocked using Block Ace (Dainippon Pharmaceutical, Osaka, Japan). Subsequently, the membrane was incubated with the following primary antibodies: argpyrimidine (ARP; 1:2000) (MMC-030n; Abcam, Cambridge, UK), Nε-(carboxymethyl) lysine (CML; 1:200) (AGE-M02; Cosmo Bio, Tokyo, Japan), Nε-(carboxyethyl) lysine (CEL; 1:400) (AGE-M01; Cosmo Bio), Nδ-(5-hydro-5-methyl-4-imidazolon-2-yl)ornithine (MG-H1) (1:1000) (STA-011; Cell Biolabs, San Diego, CA, USA), pentosidine (1:200) (PEN-12; Trans Genic Inc., Tokyo, Japan), creatine kinase (CK), mitochondrial 1A (1:2000) (Proteintech Group, Inc, Rosemont, IL, USA), and horseradish peroxidase (HRP)-conjugated anti-actin (1:10,000) (Wako Pure Chemical Industries Ltd., Osaka, Japan), diluted in Can Get Signal solution 1 (Toyobo, Osaka, Japan). The membrane was washed with PBS containing 0.1% Tween (TPBS), followed by incubation with horseradish peroxidase (HRP)-conjugated goat anti-mouse IgG (1:10,000) (Vector Laboratories, Burlingame, CA, USA) secondary antibodies diluted in Can Get Signal solution 2 (Toyobo, Osaka, Japan). The protein bands were detected using Luminata™ Crescendo western HRP substrate (EMD Millipore, Billerica, MA, USA) in a ChemiDoc Touch Imaging System (Bio-Rad Laboratories, Tokyo, Japan).

### 2.5. Liquid Chromatography-Tandem Mass Spectrometry (LC-MS/MS) Analysis

Protein identification by peptide mass fingerprinting was performed as previously described [19]. Briefly, 100 μg of protein was separated by two-dimensional polyacrylamide gel electrophoresis (2D-EP) and the gels were stained with Coomassie Brilliant Blue R-250 (CBB). Protein spots were excised from the gel and de-stained with a 50% acetonitrile solution containing 100 mM ammonium bicarbonate to remove the CBB. The de-stained gels were dehydrated using 100% acetonitrile and vacuum-dried. Subsequently, the gel was reduced with 25 mM dithiothreitol and alkylated with 54 mM iodoacetamide in 25 mM ammonium bicarbonate. Each gel was trypsinized for 3 h at 42 °C in 50 mM ammonium carbonate buffer, and the supernatant was retained. Peptides were extracted from the excised gel portions with 2.5% trifluoroacetic acid by shaking for 15 min and concentrated under vacuum. After digestion, the resultant peptides were extracted with a solution of 50% acetonitrile and 5% trifluoroacetic acid, desalted, and concentrated using StageTips, which were self-made using a solid-phase extraction disk (Empore™ 2215(FF)-C-18 Fast Flow Disk, 47 mm, 3M Company, Maplewood, MN, USA).

Peptides were analyzed using the quadrupole-Orbitrap tandem mass spectrometry (MS) system, Orbitrap Q Exactive MS connected to EASY-nLC 1000 (Thermo Fisher Scientific, San Jose, CA, USA). For peptide isolation using the nano-LC system, Acclaim PepMap 100 (75 μm × 2 cm, C18, 3 μm, 100 Å; Thermo Fisher Scientific) and NANO-HPLC capillary columns (C18, 0.1 × 125 mm; Nikkyo Technos, Tokyo, Japan) were used as the pre-column and reverse-phase analytical column, respectively. After equilibration of the columns with mobile phase A (water containing 0.1% formic acid), samples were loaded onto the pre-column at a flow rate of 300 nL/min. The gradient program using mobile phases A and B (acetonitrile containing 0.1% formic acid) had the following sequence: 0% B (0 min)–30% B (15 min)–100% B (16 min)–100% B (20 min). LC-MS/MS data were processed with Proteome Discoverer version 1.4.1.14 (Thermo Fisher Scientific) and subjected to database searching using the embedded Sequest HT server. For the identification of protein and post-translational modifications, the data were searched against human canonical sequences in the Uniprot database (version 04/2017, with 20,198 sequences).

### 2.6. Immunoprecipitation Analysis

Extracts from HIP of Wild/VB6(+) and KO/VB6(−) mice were immunoprecipitated with an antibody against mitochondrial creatine kinase (CK-mit) and protein-A-conjugated magnetic beads. A fraction of HIP extracts was directly resolved on a 5–20% gradient gel (input sample). Approximately 2.0 μg of the anti-CK-mit antibody was added to 100 μL of HIP extracts and incubated at 4 °C for 12 h, followed by the addition of 40 μL of 10 mg/mL protein-A-conjugated magnetic beads to the reaction mixture, and mixing at room temperature for 1 h to generate the protein-A-antibody conjugate. An equal amount of non-specific mouse IgG was used for the immunoprecipitation assay. The magnetic beads containing the protein-A-antibody-antigen conjugate were mixed with gel loading buffer, incubated at 90 °C for 3 min, and centrifuged to collect the supernatant. 

For Western blot analysis, these immunoprecipitation samples were resolved on a 5–20% gradient gel, transferred to PVDF membranes, and probed for MG-H1 using a mouse monoclonal antibody against MG-H1 and a goat anti-mouse horseradish peroxidase-coupled secondary antibody, as described in Section 2.4. For LC-MS/MS analysis of MG-H1-modification in CK-mit, the immunoprecipitation samples were resolved on a 12% gel, stained with CBB, and in-gel digestion of CK-mit, followed by extraction of peptides from the excised gel portions and analyzed using the quadrupole-Orbitrap tandem mass spectrometry (MS) system connected to nano-LC, as described in Section 2.5.

### 2.7. Measurement of Creatine Kinase (CK) Activity in HIP Homogenates 

Creatine kinase (CK) activities in mouse HIP of Wild/VB6(+) and KO/VB6(−) mice were determined using the corresponding assay kits (Catalog # K777-100, BioVision, Inc. Milpitas, CA, USA) according to the manufacturer’s protocols. Briefly, the supernatant of 10% HIP homogenates (1 μL) and CK assay buffer were added per well (96-well plates) to adjust the final volume to 50 µL. The reconstituted reagents (enzyme mix, color developer, and ATP solution) and substrate solution were added to start the reaction. Absorbance at 450 nm was recorded for 30 min at 37 °C using a plate reader (EnSpire, PerkinElmer, Inc. Winter Street, Waltham, MA, USA).

### 2.8. Statistical Analysis

Values are presented as mean ± standard deviation (SD). Differences between two groups were analyzed by a Student’s t test. Differences between four groups were analyzed by one-way analysis of variance (ANOVA) followed by a Tukey’s post hoc test for multiple comparisons. A *p*-value of less than 0.05 was considered statistically significant.

## 3. Results

### 3.1. Accumulation of Various AGE-Modified Proteins in Six Different Regions of the KO/VB6(−) Mice Brain 

**Figure 1:**
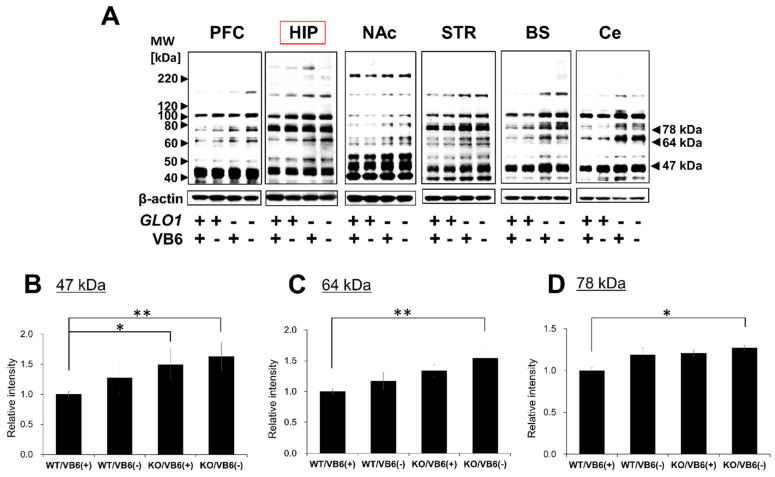
Detection of MG-H1-modified proteins in various regions of mice brain. (**A**) MG-H1-modified protein levels detected by Western blot analysis in six regions of the brain obtained from four mice experimental groups. (**B**–**D**) Densities of (**B**) 47 kDa, (**C**) 64 kDa, and (**D**) 78 kDa bands from HIP regions were measured and each ratio to β-actin was calculated and expressed as fold-change of the band intensity measured in WT/VB6(+) mice. Values are presented as mean ± standard deviation (SD) (*n* = 4) (* *p* < 0.05, ** *p* < 0.01).

**Figure 2:**
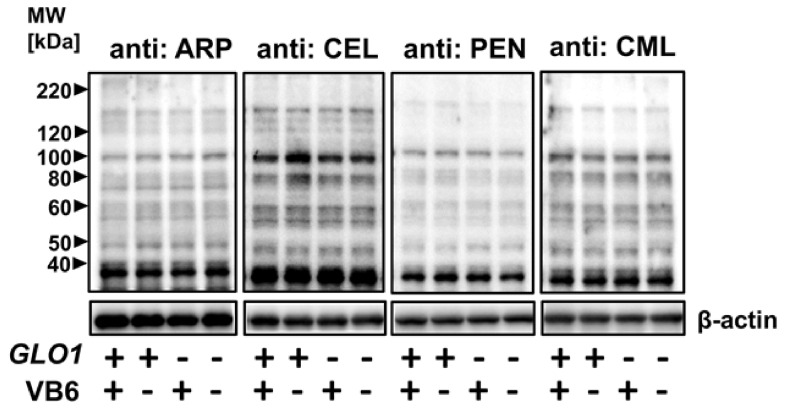
Detection of various AGEs-modified proteins in HIP. ARP, CEL, PEN, and CML-modified protein levels were detected by Western blot analysis in HIP obtained from four mice experimental groups. A typical result from the other two samples providing similar results is presented.

AGEs are generated from α-dicarbonyl compounds, such as GO and MGO [20]. Thus, it is expected that the accumulation of MGO will lead to the formation of modified proteins with glycation sites, such as MG-H1, ARP, CEL, PEN, and CML in the brain of KO/VB6(−) mice. The accumulation of carbonyl proteins was detected in six different regions of the mouse brain using Western blot analysis. As shown in Figure 1A, carbonyl proteins accumulated with the MG-H1 moiety in the PFC, HIP, STR, Ce, and BS regions of the brain in *Glo1* knockout mice. The band intensities of 47 kDa (Figure 1B), 64 kDa (Figure 1C), and 78 kDa (Figure 1D) proteins with MG-H1 were significantly increased in the HIP region of the brain in KO/VB6(−) mice, when compared to Wild/VB6(+) mice (*n* = 4, Supplementary Figure S1). Western blot analysis using CEL and ARP antibodies showed slightly increased band intensity in six brain regions (Supplementary Figure S2A–D), including the hippocampus (Figure 2), in KO/VB6(−) mice. However, the reproducibility of the results was insufficient, and there was no significant difference between the band intensities of the KO/VB6(−) and Wild/VB6(+) mice. Additionally, various bands were detected by the CML antibody, making it unclear whether this was due to low specificity or the presence of many endogenous CML-modified proteins. In any event, there was no significant difference between the hippocampus of the KO/VB6(−) and Wild/VB6(+) mice.

### 3.2. Identification of Protein Modified with MG-H1 Moiety in the KO/VB6(−) Mice Brain 

**Figure 3:**
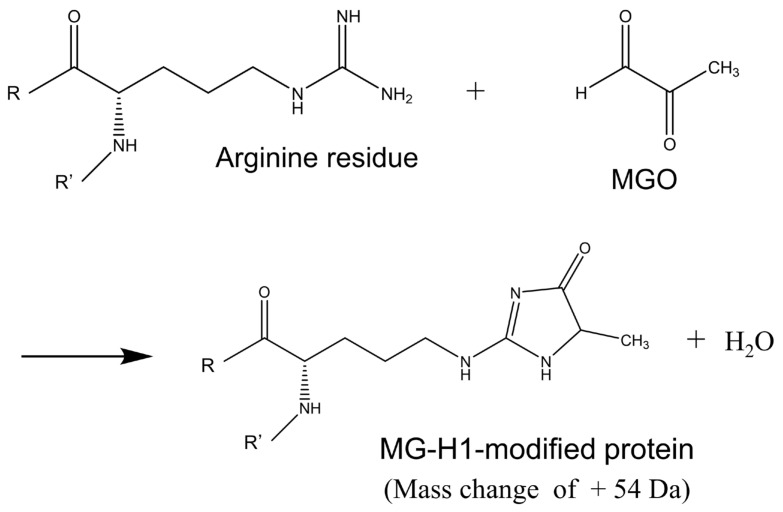
Formation of MG-H1 moiety with the reaction between MGO and arginine residue in the polypeptide.

**Figure 4:**
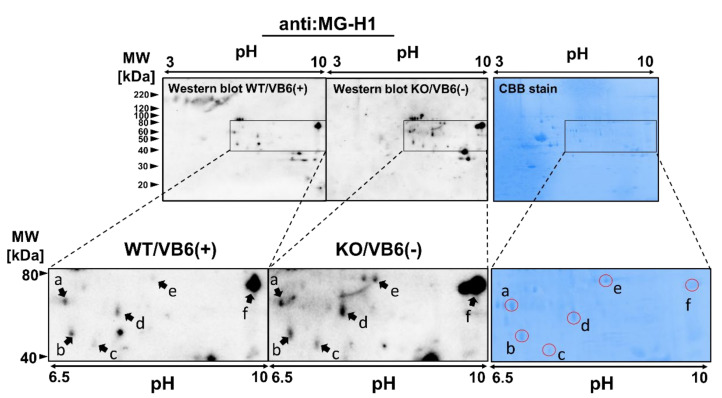
Identification of MG-H1-modified proteins in the HIP from KO/VB6(−) mice. HIP homogenates of WT/VB6(+) mice or KO/VB6(−) mice were separated by two-dimensional gel electrophoresis. MG-H1-modified proteins were identified by Western blot analysis using anti-MG-H1 antibody. The left and middle panels show MG-H1-modified proteins detected by Western blot analysis after two-dimensional gel electrophoresis in the HIP of WT/VB6(+) mice and KO/VB6(−) mice, respectively. A typical result from the other two samples providing similar results is presented. HIP samples of WT/VB6(+) mice or KO/VB6(−) mice were subjected to two-dimensional gel electrophoresis followed by CBB staining. The right panel shows a two-dimensional gel image with the CBB staining pattern. The specified spots (spot a, b, c, d, e, and f) were excised and digested in-gel for LC-MS/MS analysis.

The three bands detected as MG-H1-modified proteins at 47, 64, and 78 kDa were significantly increased in the HIP of KO/ mice brain. Thus, we attempted to identify MG-H1-modified protein contained in the three bands by nano-LC-MS/MS analysis with a database including a modification library. As shown in Figure 3, the MG-H1 moiety in the protein was derived from an arginine residue by the attachment of MGO with a concomitant loss of a single water molecule (mass change of +54 Da). However, due to the overabundance of protein impurities in each excised band, proteins with different modification characteristics between the KO/VB6(−) and Wild/VB6(+) mice could not be determined. Therefore, the same experiment was performed after conducting two-dimensional electrophoresis (2D-EP) on hippocampus samples prepared from four KO/VB6(−) and four Wild/VB6(+) mice. As shown in Figure 4, we focused on six protein spots (a (68 kDa), b (54 kDa), c (47 kDa), d (63 kDa), e (84 kDa), and f (80 kDa)), which showed higher intensities than those in WT/VB6(+) mice, when probed with the anti-MG-H1 antibody in HIP samples from KO/VB6(−) mice. Based on the results shown in Figure 4, in order to identify the MG-H1-modified proteins, these six CBB-stained spots (a, b, c, d, e, and f) were subjected to in-gel digestion by trypsin and nano-LC-MS/MS analysis, respectively. A comparison of the results for six protein spots (Figure 4) obtained by LC-MS/MS analysis with data processing, including post-translational modifications, indicated the presence of multiple polypeptides containing MG-H1-modified arginine residues (Figure 3) in the hippocampus of the four KO/VB6(−) mice; however, none were detected in the hippocampus of the four Wild/VB6(+) mice. Seven different MG-H1-modified proteins were identified when the results of LC-MS/MS were analyzed using the database to assign MG-H1-modified polypeptides (Table 1).

As shown in Table 1, we found T-complex protein 1 and Tubulin alpha-4A chain from spot a, gamma enolase from spot b, mitochondrial creatine kinase (CK-mit) from spot c, collapsin response mediator protein 4 (CRMP4) from spot d, beta-adrenergic receptor kinase 1 (GRK2) from spot e, and Synapsin-1 from spot f, as the levels of MG-H1-modified proteins were increased by *Glo1*-KO and VB6 deficiency in the HIP.

### 3.3. Immunoprecipitation Study for Quantitative Estimation of the MG-H1-Modified Proteins

**Figure 5:**
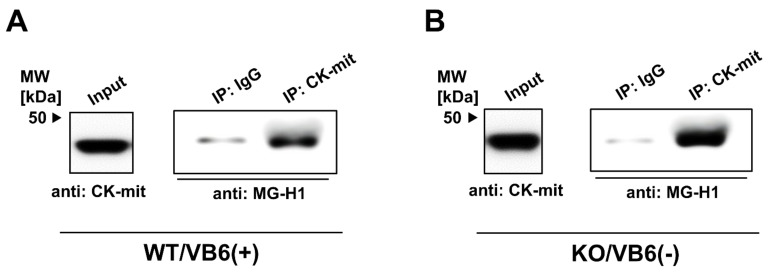
Quantitative detection of MG-H1-modified U-type Creatine kinase by immunoprecipitation. Proteins extracted from the HIP of WT/VB6(+) (**A**) or KO/VB6(−) (**B**) mice were immunoprecipitated with mouse IgG or mouse antibody against CK-mit (**A**,**B**). HIP homogenate (Input) was loaded as a control. Each sample recovered was electrophoresed by SDS-PAGE (5–20% gradient gel) and immunoblotted with the antibody against MG-H1. A typical result from duplicate experiments is presented.

As LC-MS/MS does not provide quantitative information on the MG-H1-modified proteins, CK-mit from the HIP fraction of WT/VB6(+) mice or KO/VB6(−) mice was immunoprecipitated using specific antibodies, with MG-H1-modified protein levels determined by Western blot analysis. A distinct increase in MG-H1 modification of CK-mit was observed in KO/VB6(−) mice, compared with WT/VB6(+) mice (Figure 5). The quantification of band intensities (MG-H1/input) indicated that relative MG-H1 modification ratio of CK-mit in KO (-) mice were two times higher than that in WT (+) mice. We also performed immunoprecipitation for these candidate proteins (T-complex protein 1, GRK2). However, none of their levels were clearly increased in GLO1 KO mice compared with WT mice (data not shown).

### 3.4. Intensive Comparison of Mitochondrial Creatine Kinase in Hippocampus between KO/VB6(−) and Wild/VB6(+) Mice 

We focused on MG-H1 modification of CK-mit because CK is specifically modified by phenylglyoxal on arginine residues with inactivation [21]. To clarify more comprehensively the MG-H1-modification status of arginine residues in CK-mit from HIP, the purified CK-mit was subjected to LC-MS/MS analysis. CK-mit was purified from HIP of KO/VB6(−) or Wild/VB6(+) mice by immunoprecipitation using anti-CK-mit antibody followed by SDS-PAGE and in-gel digested with trypsin, and then the generated peptides were analyzed using LC-MS/MS. Table 2 shows the MG-H1 modifications of CK-mit from HIP of KO/VB6(−) mice. A database search confirmed the identification of CK-mit (P30275) with sequence coverage of 69.38%, higher than that of 39.23% obtained by the analysis of total proteins (Table 1). Furthermore, in addition to R400 and R403, it was newly found that nine arginine residues (R12, R16, R33, R46, R58, R75, R164, R166, R169) were modified with MG-H1 moiety (Table 2). In contrast, very few arginine residues were MG-H1-modified in the purified CK-mit from HIP of the Wild/V6(+) mice. From these results, it was indicated that the MG-H1-modification of arginine residues in CK-mit was clearly enhanced in HIP of KO/VB6(−) mice.

**Figure 6:**
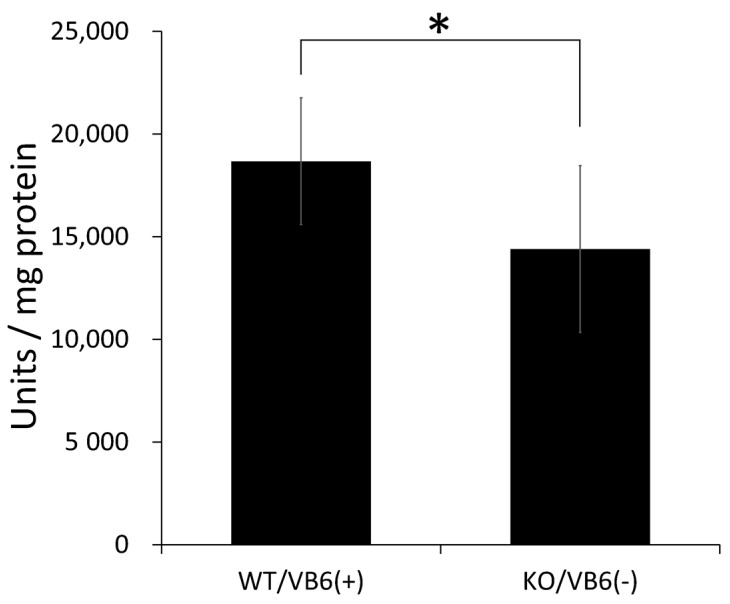
Creatine kinase activity in mouse hippocampus homogenates. CK activity (U/mg) was assayed using hippocampal homogenates (20 g) of WT/VB6(+) mice or KO/VB6(−) mice. Values are presented as mean ± SD (*n* = 8). Error bars represent SD. * *p* < 0.05.

Moreover, we examined the effect of the MG-H1-modification on CK activity in the HIP region. When CK activities were assayed in hippocampal homogenates prepared from KO/VB6(−) and Wild/VB6(+) mice, a significant decrease in CK activity was observed in the hippocampus of KO/VB6(−) compared to that of Wild/VB6(+) mice (Figure 6).

## 4. Discussion

MGO, a highly reactive dicarbonyl compound produced during glucose metabolism, chemically modifies proteins and generates a dicarbonyl proteome [20]. Although it is not clear whether MGO levels in CS-SCZ patients are higher than those in healthy subjects, we have previously shown that MGO-induced carbonyl protein (ARP-modified protein) levels in some CS-SCZ patients are significantly higher than those in healthy subjects [22]. Thus, the present study focused on the accumulation of MGO-induced carbonyl proteins in the brain of KO/VB6(−) mice. 

Previous studies have reported that the functional and morphological abnormalities associated with the PFC, STR, and HIP regions of the brain are related to SCZ symptoms [23,24,25,26,27]. In particular because of the abnormality of HIP and memory impairment found in patients with SCZ, many studies have suggested that the dysfunction of HIP is directly related to SCZ pathophysiology [28,29,30]. Hence, the restoration of hippocampal function in patients is essential for the treatment of SCZ. However, the mechanism of impairment of this function is not yet fully understood, and thus, an effective therapy for SCZ patients has not been developed. We previously reported that MGO is a potent neurotoxic agent that, when present in excess levels in neurons, can react with the lysine and arginine residues of proteins [31]. The present study evaluated the state of carbonyl stress in KO/VB6(−) mice as a CS-SCZ model by examining the changes in carbonyl protein with AGEs in the brain, in order to investigate the mechanism of SCZ pathogenesis.

In the present study, we found the accumulation of carbonyl proteins with the MG-H1 moiety in five regions of the brain in Glo1 knockout mice (Figure 1A). In particular, many MG-H1-modified proteins were significantly increased in the HIP region of the brain in KO/VB6(−) mice, when compared to Wild/VB6(+) mice. We focused on the hippocampus in the brain due to its close relationship with schizophrenia and investigated MG-H1-modified proteins accumulated in the hippocampus of KO/VB6(−) mice. Although Arai et al. have previously reported that carbonyl proteins with a PEN moiety show significant accumulation in the plasma of CS-SCZ patients [2], a significant increase in the band intensities corresponding to PEN-modified proteins was not observed in the brains of KO/VB6(−) mice. These results suggested that MG-H1-modified protein levels might also be higher in the brains of CS-SCZ patients at the early stage of SCZ onset. It has been reported that MG-H1 may act as a marker of the early stages of arteriosclerosis in childhood diabetes [32]. Meanwhile, it has been reported that there is a weak correlation between pentosidine level and joint damage in early-stage osteoarthritis [33]. Additionally, the specific accumulation of MG-H1-modified proteins has been reported in cases of kidney failure [34] and multiple sclerosis [35]. Also, we showed that carbonyl protein levels including the MG-H1 moiety in plasma were higher in younger CS-SCZ patients using the 2,4-dinitrophenylhydrazine (DNPH) method [36]. These results may indicate that MG-H1-modified protein accumulates in the primary stage of CS-SCZ because of MG-H1-modified protein having a keto group detectable by the DNPH method, whereas the PEN-modified protein without the keto group cannot be measured by DNPH derivatization. Therefore, the histochemical studies based on the present results are particularly desired to show the localization of MG-H1-modified proteins in the brain comparing genotype/food of model mouse. Moreover, we propose the quantification of the complete array of AGEs and total carbonyl proteins to estimate the state of carbonyl stress in patients with SCZ.

It is believed that CEL, ARP, and MG-H1 are primarily derived from MGO and proteins in mammalian tissues [37]. In this study, a significant accumulation of MG-H1-modified proteins was observed in the KO/VB6(−) mice groups (Figure 1). Itokawa et al. reported that in clinical trials, treatment with excess VB6 ameliorated SCZ symptoms [13]. Therefore, our results seem to indicate that VB6 deficiency as well as Glo1 knockout are key factors for the accumulation of MG-H1-modified proteins. Further studies are required to understand the combined effects of Glo1 knockout and VB6 deficiency on the brain to elucidate the mechanism of CS-SCZ onset.

In the present study, we also found that accumulation of MG-H1 modified CK-mit and a significant decrease in CK activity in the hippocampus of KO/VB6(−) mice. It is well-known that inactivation of CK is induced by modification of its arginine residues with phenylglyoxal [38] and MGO [39]. CK catalyzed the reversible reaction, converting creatine and ATP to phosphocreatine and ADP. Two types of CK, cytosolic brain-type creatine kinase and ubiquitous CK-mit, are expressed in the mammalian brain and are found in abundance in the HIP region [40]. Cytosolic and mitochondrial CKs cooperate to maintain cellular homeostasis. However, these are easily oxidized by ROS, which decrease their activity, leading to mitochondrial dysfunction [41]. Therefore, abnormal CK function associated with neuronal diseases is mediated by oxidative stress [42]. Indeed, previous reports have indicated that a ketamine-induced SCZ rat model showed a significant increase in carbonyl protein levels and a noticeable decrease in the CK activity of the HIP region [43,44]. 

Many neuropsychiatric and neurodegenerative disorders, such as schizophrenia, depression, Alzheimer’s Disease, and Parkinsonism, can be severely functionally debilitating in nature. On the other hand, phytochemicals have a neuroprotective effect which may prove beneficial in different neuropsychiatric and neurodegenerative disorders [45]. Thus, some synergistic effects are expected because supplemental intake of selected dietary phytochemicals [46,47] not only scavenge free carbonyl groups, but also inhibit protein misfolding, oxidative stress, mitochondrial damage, and neuronal apoptosis. 

## 5. Conclusions

In conclusion, our findings revealed that Glo1-KO with VB6 deficiency leads to a significant accumulation of MG-H1-modified proteins, in the HIP, PFC, BS, STR, and Ce regions of the mouse brain. Chemically, MG-H1 is a typical AGE derived as a result of the Maillard reaction by MGO on proteins. Thus, it is plausible that excess MGO induces MG-H1 modification in specific proteins, such as CK-mit, GRK2, and T-complex proteins in the mouse brain. Our previous study demonstrated that carbonyl proteins accumulated in the plasma of CS-SCZ patients [36]. In the present study, we found that carbonyl proteins were specifically accumulated in the brain of KO/VB6(−) mice, which may be a CS-SCZ model. Moreover, our observation of a significant inactivation of CK in the hippocampus from KO/VB6(−) mice indicates a possibility that the dysfunction of CK-mit is involved in the development of CS-SCZ. It is expected that future research will shed light on the structural and functional changes of MG-H1-modified proteins, including CK-mit, in the brain of KO/VB6(−) mice, in order to elucidate the mechanism of CS-SCZ onset.

## Figures and Tables

**Table 1:** List of MG-H1-modified proteins in HIP of KO/VB6(−) mice #, identified with LC-MS/MS analysis.

Spot	Accession No.	Protein Name	Coverage	Peptide Identification	Modified Residue
a	P80318	T-complex protein 1	55.23%	GASKEILSEVER*	R389
	P68368	Tubulin alpha-4A chain	32.14%	CDPR*HGKYMACCLLYR*	R308, R320
b	P17183	Gamma enolase	30.41%	QR*YLGKGVLK	R56
c	P30275	Creatine kinase U-type, mitochondrial	39.23%	R*LER*GQDIR_403_	R400, R403
d	O35098	CRMP4	32.69%	VVLEDGNLLVTPGAGR*	R467
e	Q99MK8	Beta-adrenergic receptor kinase 1	27.00%	YFYLFPNRLEWR*	R591
f	O88935	Synapsin-1	69.69%	QR*QAGAPQATR*	R556, R565

* MG-H1 modification, # *n* = 3.

**Table 2:** LC-MS/MS analysis of MG-H1 modification in mitochondrial Creatine kinase purified from HIP of KO/VB6(−) mice #.

Accession No.	Protein Name	Coverage	Peptide Identification	Modified Residue
P30275	Creatin kinase U-type, mitochondrial	69.38%	_1_MAGPFSRLLSAR*PGLR*LLALAGAGSLT AGILLR*PESVGAAAAER_44_	R12, R16, R33
			_46_R*LYPPSAEYPDLR*K_59_	R46, R58
			_45_RRLYPPSAEYPDLRKHNNCMASHLTPAV YAR*LCDKTTPTGWTLDQCIQTGVDNPGHPFIK_104_	R75
			_152_SGYFDERYVLSSR*VR*TGR*_169_	R164, R166, R169
			_400_R*LER*GQDIR_408_	R400, R403

* MG-H1 modification, # *n* = 3.

## Data Availability

Not applicable.

## Supplementary Materials

The following are available online at https://www.mdpi.com/article/10.3390/antiox10040574/s1, Figure S1; Detection of MG-H1-modified proteins in mouse HIP (*n* = 4). Figure S2: Detection of various carbonyl proteins modified by AGEs in various regions of mice brain.
