# Supplementary material for: Accumulation of Carbonyl Proteins in the Brain of Mouse Model for Methylglyoxal Detoxification Deficits
Source: Antioxidants (Basel). 2021 Apr 8;10(4):574. doi: 10.3390/antiox10040574 (PMC8068291; doi:10.3390/antiox10040574)

Supplemental Figure 1

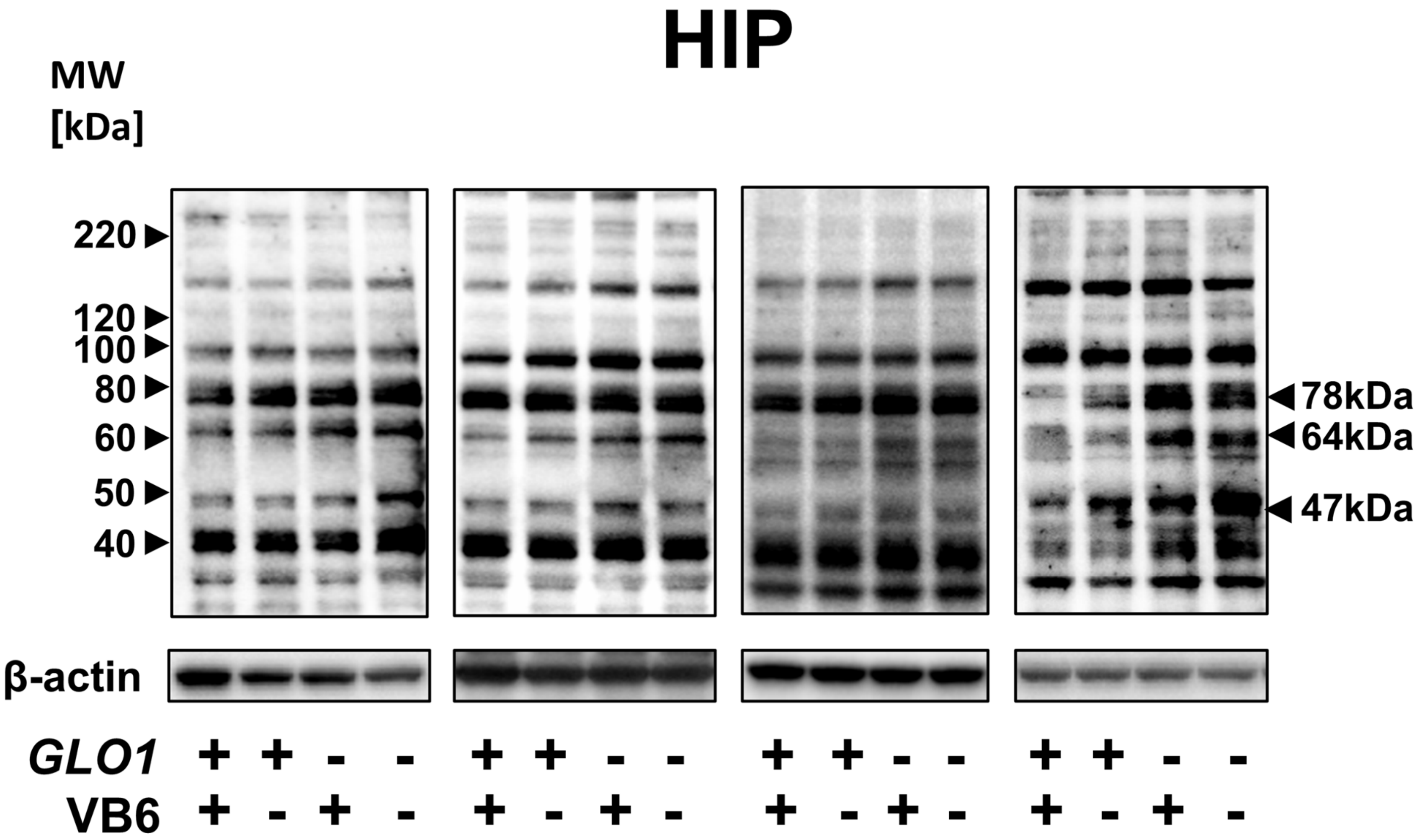

Supplemental Figure 2A

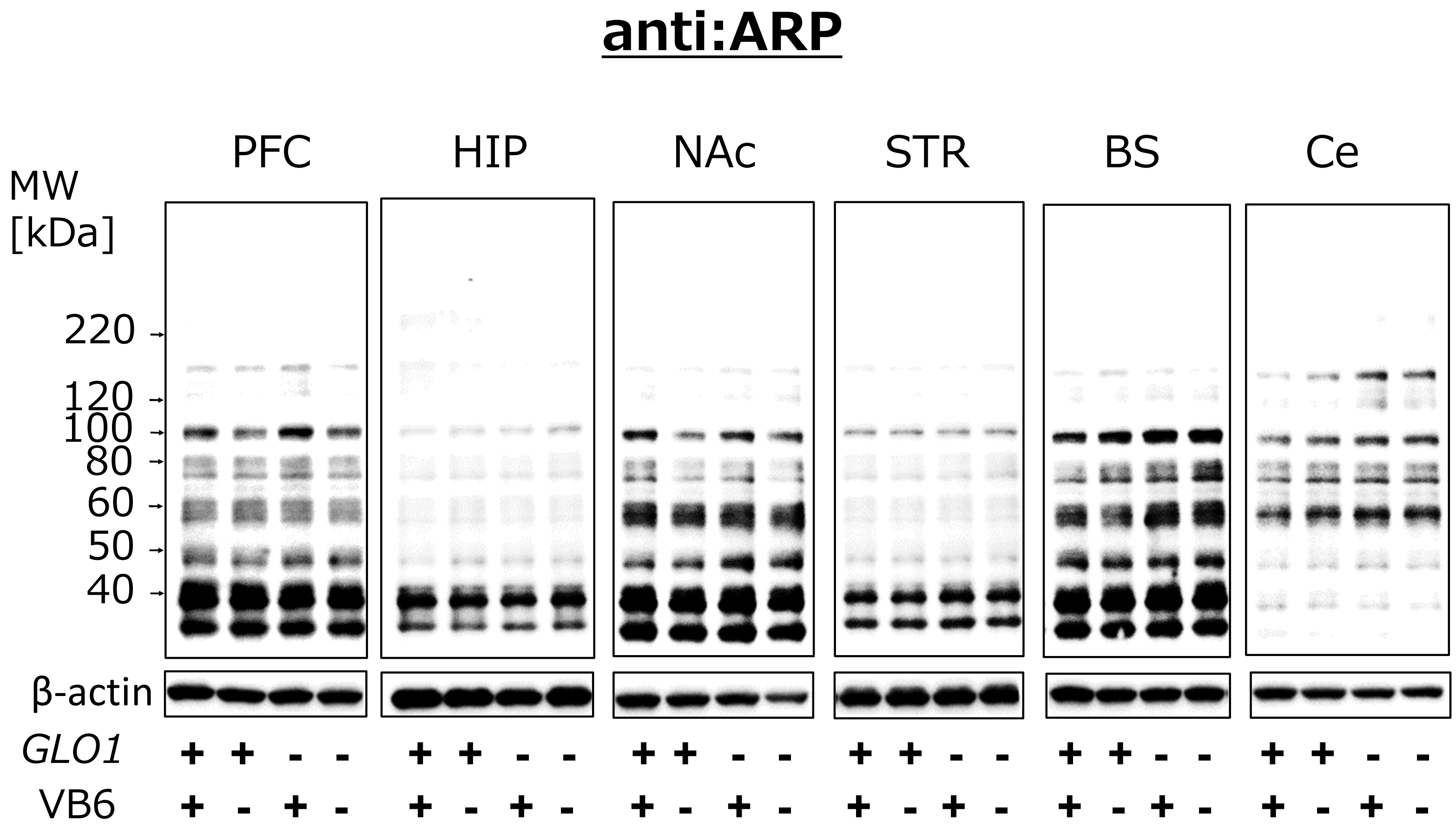

Supplemental Figure 2B

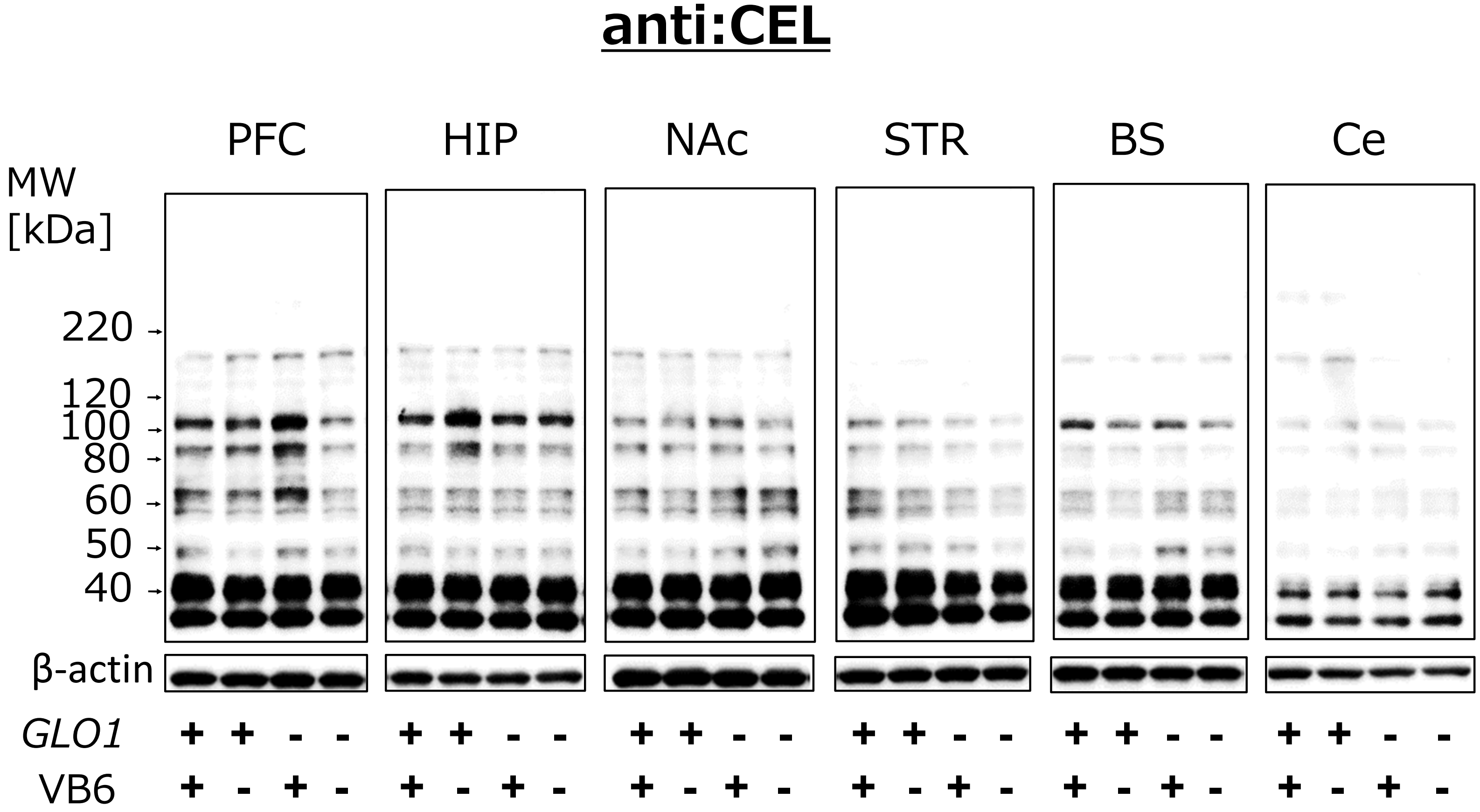

Supplemental Figure 2C

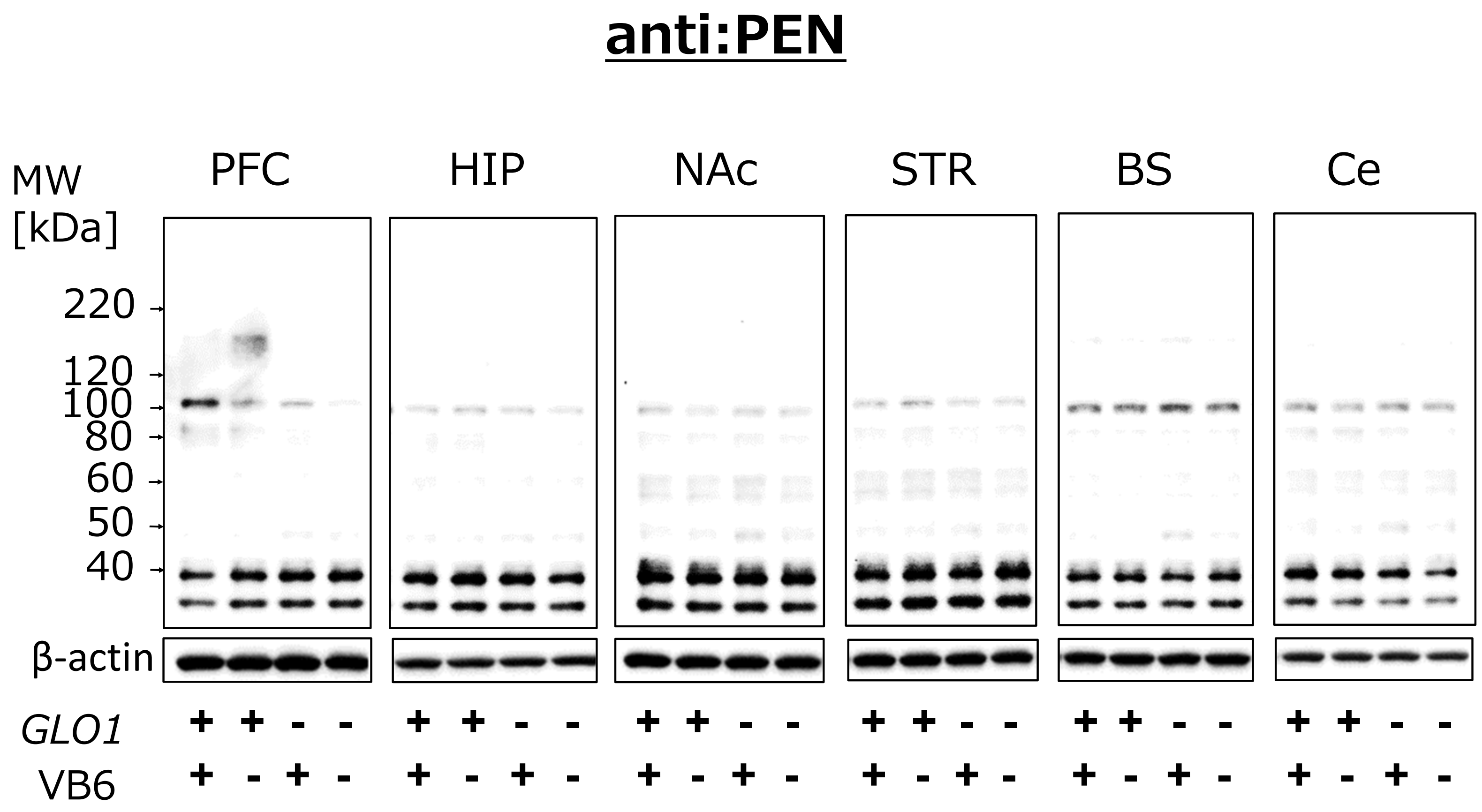

Supplemental Figure 2D

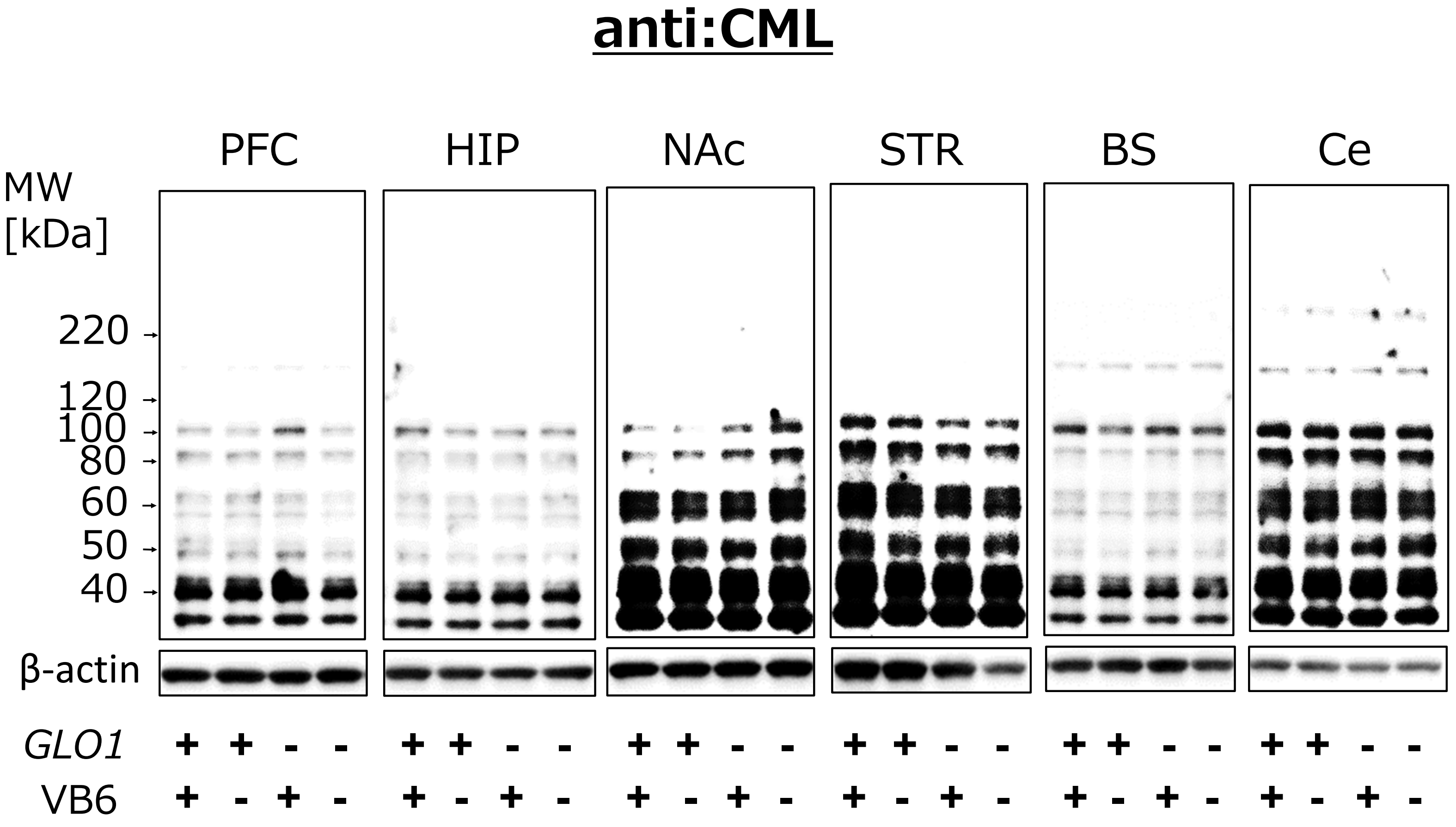

Supplement: Supplementary file 1 [file antioxidants-10-00574-s001.pdf]
